# The Bioavailability of Drugs—The Current State of Knowledge

**DOI:** 10.3390/molecules28248038

**Published:** 2023-12-11

**Authors:** Marlena Stielow, Adrianna Witczyńska, Natalia Kubryń, Łukasz Fijałkowski, Jacek Nowaczyk, Alicja Nowaczyk

**Affiliations:** 1 Bonkitel, 1A/14 Staszica Street, 85-014 Bydgoszcz, Poland; biuro@bonkitel.com; 2 Department of Organic Chemistry, Faculty of Pharmacy, Collegium Medicum in Bydgoszcz, Nicolaus Copernicus University, 2 Jurasza Street, 85-089 Bydgoszcz, Poland; adrianna.witczynska@doktorant.umk.pl (A.W.); natalia.kubryn@doktorant.umk.pl (N.K.); l.fijalkowski@cm.umk.pl (Ł.F.); 3 Department of Physical Chemistry and Physicochemistry of Polymers, Faculty of Chemistry, Nicolaus Copernicus University, 7 Gagarina Street, 87-100 Toruń, Poland; jacek.nowaczyk@umk.pl

**Keywords:** bioavailability, pharmacokinetics, in vitro and in vivo evaluation methods, therapeutic effectiveness, pharmaceutical innovations

## Abstract

Drug bioavailability is a crucial aspect of pharmacology, affecting the effectiveness of drug therapy. Understanding how drugs are absorbed, distributed, metabolized, and eliminated in patients’ bodies is essential to ensure proper and safe treatment. This publication aims to highlight the relevance of drug bioavailability research and its importance in therapy. In addition to biochemical activity, bioavailability also plays a critical role in achieving the desired therapeutic effects. This may seem obvious, but it is worth noting that a drug can only produce the expected effect if the proper level of concentration can be achieved at the desired point in a patient’s body. Given the differences between patients, drug dosages, and administration forms, understanding and controlling bioavailability has become a priority in pharmacology. This publication discusses the basic concepts of bioavailability and the factors affecting it. We also looked at various methods of assessing bioavailability, both in the laboratory and in the clinic. Notably, the introduction of new technologies and tools in this field is vital to achieve advances in drug bioavailability research. This publication also discusses cases of drugs with poorly described bioavailability, providing a deeper understanding of the complex challenges they pose to medical researchers and practitioners. Simultaneously, the article focuses on the perspectives and trends that may shape the future of research regarding bioavailability, which is crucial to the development of modern pharmacology and drug therapy. In this context, the publication offers an essential, meaningful contribution toward understanding and highlighting bioavailability’s role in reliable patient treatment. The text also identifies areas that require further research and exploration.

## 1. Introduction

Drug bioavailability plays a crucial role in the effectiveness of pharmacological therapy. It determines the degree and rate at which drug-active substances are absorbed into the bloodstream after oral, topical, parenteral, and rectal administration [1]. In practice, bioavailability indicates the amount of the administered dose of a drug reaching the bloodstream in the form of the active ingredient which is then available to the body to produce a therapeutic effect [2]. 

Bioavailability is affected by variety of factors, including the medication’s physicochemical properties, the mode of administration, interactions with other substances, absorption, hepatic metabolism, and excretion [3,4]. The bioavailability of the active pharmaceutical ingredients (API) corresponds to the dose entering the bloodstream and consequently, its effectiveness [2]. For this reason, a drug’s bioavailability must be considered when designing a therapy and dosage. The efficient adjustment of an administered dosage requires knowledge of the API’s absorption, transport mechanism, metabolism, and elimination from the system [5]. Conversely, the ineffective administration of medications adds to the escalation of superfluous drug use. From this perspective, the significance of bioavailability becomes even more significant when it comes to patient safety and treatment efficacy [6]. Improving medication bioavailability can be accomplished by using suitable drug delivery methods, modifying drug formulations, optimizing dosages, identifying and controlling factors that decrease bioavailability, and monitoring blood drug levels resulting from dose adjustments. The monitoring of bioavailability can provide insights into drug interactions, as well as enable the development of tailored treatment plans for patients with liver or intestinal dysfunction [7]. Safe drug therapy requires good bioavailability. Insufficient bioavailability can reduce therapy efficacy, whereas excessive medication concentrations can produce toxicity and side effects [8]. Nevertheless, drug bioavailability is only one of many factors impacting the efficacy of drug therapy [9]. Currently, the field of pharmaceutical research and development is facing various challenges, one of which is the optimization of medication bioavailability. This optimization is pursued with the goal of enhancing the safety and efficacy of treatments [10].

The aim of this study is to acquaint the reader with the general aspects of the bioavailability of drugs and their importance in the context of drug safety and therapy strategies. During the last few years, a plethora of papers have been published on bioavailability. However, these involve quite specific cases or analyze pharmacokinetic mechanisms from a limited perspective. Since, over the last 5 years, there has been a lack of works published providing a general description of recent findings and a comprehensive discussion of the problem as a whole, the authors intend to present such a study to the audience. The novelty of our review comes from a comprehensive generalization of the bioavailability problem, particularly in the context of drug safety.

This publication explores drug bioavailability, a crucial indicator of pharmacological therapy efficacy. It explains basic concepts and measures, including the release, absorption, distribution, metabolism, and elimination of drugs [11]. Factors affecting bioavailability include physicochemical, biological, pharmaceutical, and patient-related aspects. Methods for assessing bioavailability are discussed, including in vitro and in vivo studies. New technologies and tools are used in bioavailability research, such as mathematical models, in silico simulations, nanotechnology, and genetically targeted therapies. The importance of drug bioavailability research is highlighted for improving therapy effectiveness, personalizing treatment, minimizing side effects, and developing new drugs and formulations [12]. Bioavailability is also important for treating rare diseases, with limited effectiveness [13].

## 2. Materials and Methods

Dataset: The literature query was analyzed using PRISMA (Preferred Reporting Items for Systematic Review and Meta-Analyses) guidelines [14].

Literature databases and search pattern: The search was conducted using a combination of keywords including bioavailability, pharmacokinetics, in vitro and in vivo evaluation methods, therapeutic effectiveness, and pharmaceutical innovations. The set of databases included Science Direct, Scopus, and PubMed. Records were identified for full-text scientific articles, published only in chemistry, pharmacology, toxicology or pharmacy journals, between 2019 and 2023. We also manually searched the bibliography of selected articles, reviews, meta-analyses, and practical tips. The 125 articles selected have been mutually agreed upon by the authors. 

## 3. Bioavailability of Drugs: Basic Concepts and Controlling Factors

The bioavailability of a drug describes the level of absorption of the active substance contained therein and the speed at which it is absorbed from the form administered to the patient, becoming available in the targeted location of the body, usually in the bloodstream. The bioavailability of a drug determines the proportion of the active substance available in the body in relation to its amount in the drug [15].

**Figure 1:**
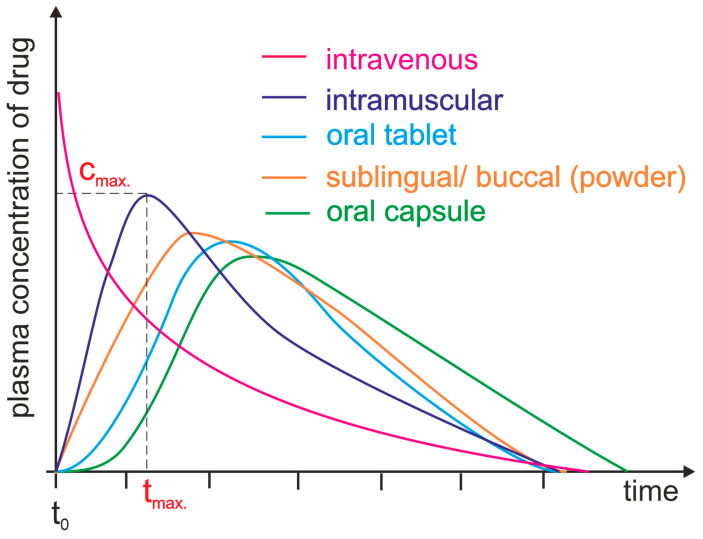
Plasma level time curves for different types of drug administration. The drug is delivered directly into the systemic circulation via intravenous injection, ensuring 100% bioavailability and immediate achievement of maximum plasma concentration (c_max_, t_max_ = 0 min). For other parenteral routes, such as subcutaneous and intramuscular injections, most drugs show between 60 and 100% bioavailability due to little or no metabolism in the skin or muscle. However, the time to reach maximum plasma concentration (t_max_) is significantly longer than that achieved by intravenous administration. Orally administered drugs achieve a bioavailability level substantially lower than 100% due to incomplete absorption and/or elimination during the first pass through the liver. Additionally, due to the indirect path to the plasma, they are characterized by a long time lag. Different dosage forms may result in differences in c_max_ and t_max_.

Drug bioavailability measurements allow for the assessment of the absorption efficiency of a drug, including its absolute and relative bioavailability, the time to reach maximum concentration, and the area under the concentration–time curve [16]. Absolute bioavailability is defined as a measurement that determines the percentage of the active substance entering the bloodstream after administration of a drug when the reference standard is an intravenous dose. It expresses the efficiency of the absorption of the active substance by the body. It is usually less than 100%, since not all of the active substance is absorbed in the gastrointestinal tract [17]. Relative bioavailability is expressed as the ratio of the bioavailability of two dosage forms of the same drug (Figure 1). We can compare the bioavailability of a drug in tablet form to its bioavailability in syrup form [18]. The time to reach maximum concentration (t_max_) measures the time it takes for an active ingredient to reach its highest concentration in the blood after drug administration. t_max_ is an important parameter because it can affect the rate of action of a drug. The area under the curve (AUC) measures the total amount of an active substance absorbed and available in the bloodstream as a function of time. The AUC is used to assess the total exposure of the body to an active substance (Figure 1) [19]. Measurements of drug bioavailability are essential for evaluating the pharmacokinetics and pharmacodynamics of drugs and for determining appropriate therapeutic doses. They are also crucial for developing new drugs and evaluating the efficacy of different administration forms [16,20].

The ADME processes affect drug absorption, distribution, metabolism, and elimination in the body [21,22].

Absorption is the passing of a drug from the administration site (e.g., gastrointestinal tract, skin, respiratory system) into the bloodstream [23]. When administered orally, the drug must be absorbed from the gastrointestinal tract into the bloodstream to achieve its effect. Various factors can determine absorption, such as dosage form, the presence of food, the environment’s pH, or interactions with other substances [24]. 

Distribution is defined as the spread of an active compound throughout the body, leading to the presence of the drug in various tissues and organs after its absorption into the bloodstream. Distribution depends on several factors, such as vascular resistance, distribution volume, degree of drug binding to plasma proteins, and penetration of tissue barriers (e.g., permeability of the blood–brain barrier) [25]. Once a substance enters the body, part of it can bind to proteins in the blood, mainly albumin (acidic and neutral molecules, such as vitamins, drugs, and their metabolites), but also other proteins, such as acidic α1-glycoprotein and lipoproteins, as well as gamma globulins. Drug–protein complexes are too large to passively penetrate cell membranes, which affects drug distribution in the body. For the desired pharmacological effect to be achieved, most of the drug must be in free (unbound) form, since they can only interact with receptors in this form. If two or more drugs bind to the same plasma protein, they compete for a binding site, affecting their biological availability, as well as their effects [26]. A number of plasma proteins in the human body, such as albumin (HSA) and al-fa-1-acid glycoprotein (AGP), enable the binding of drugs and contaminants. HSA and AGP play a significant role in transferring and storing various substances in the bloodstream. They are also used as biomarkers of inflammation and liver disease. Both proteins can transport endogenous substances, i.e., serotonin or histamine, and exogenous substances, including drugs and pollutants [27]. AGP is classified as a positive acute-phase protein [28]. The body’s first line of defense against the disruption of homeostasis is the onset of inflammation, which causes an increase in AGP concentrations. AGP has many functions, such as inhibiting lysosomal and proteolytic enzyme activity, activating the complement system, participating in blood clotting by inhibiting or enhancing platelet aggregation, and binding and neutralizing pathogens. In a healthy adult, the AGP concentration in serum ranges from 0.55 to 1.40 g/L. Under pathological conditions, the concentration of AGP increases by at least 25% [26]. The single polypeptide chain of serum AGP (183 residues, average Mw ≈ 41,000) is highly glycosylated, and the carbohydrate content is 41–45%. The acidity of the protein (isoelectric point = 2.7) is due to the presence of sialic acid residues. The genetic polymorphism of AGP has long been known, and it exhibits three main variants (F1, S, and A), which differ in primary structure. The difference between F1 and S is due to the substitution one amino acid, while the A variant differs from F1/S in 22 residues [29]. HSA is the most abundant carrier protein in plasma, with a concentration of 0.6 mM. It is versatile and non-glycosylated, with multiple functions [30]. The physiological concentration of serum albumin is about 50 times higher than that of AGP; hence, more of the drug is bound to HSA by volume for drugs that bind to both albumin and AGP [26]. The impressive capability of HSA lies in its reversible binding and transportation of a wide range of hydrophilic and negatively charged small molecules during circulation [31]. Albumin has various functions, meaning that a decrease in albumin concentration significantly impacts tissue fluid distribution, metabolism, nutrition, and substrate transport. HSA regulates human physiology, including regulating the redox potential of the colloidal osmotic pressure of the plasma between the blood and tissues. It is responsible for 75% of normal oncotic pressure [31]. The decrease in the concentration of HSA in the plasma can lead to metabolic alkalosis. The albumin molecule contains multiple charged amino acid residues that act as a buffer in the plasma. At a pH level considered normal for the body, HSA has a negative charge. Besides improving drug solubility, binding to albumin is also helpful in reducing toxicity and prolonging the drug’s half-life. HSA plays a vital role in enhancing the solubility of hydrophobic drugs, transporting them to specific organs and tissues, or removing them when harmful [32]. Biotechnological methods that use the long half-life of albumin in circulation can help improve the pharmacokinetics and biodistribution of drugs, therapeutic proteins, and nucleic acids [31].

The process by which the body, with the assistance of enzymes, transforms a drug into different substances—known as metabolites—is referred to as drug metabolism [33]. The liver is the most critical site of drug metabolism. This process aims to convert the drug into more soluble substances, which are more efficiently eliminated from the body, and to manage its concentration and activity [34]. 

Eliminating drugs from the body involves the removal of both the drug and its metabolites from the organism. The two main routes of elimination are excretion through urine and secretion via bile. The pharmacokinetic processes play a vital role in determining the appropriate drug doses, determining the optimal timing of administration, and assessing the therapeutic effectiveness. [19].

Factors influencing the bioavailability of drugs can be divided into four main categories: physicochemical agents, biological agents, pharmaceutical agents, and patient factors. The physicochemical properties of a drug are important for its bioavailability. Examples of physicochemical factors include drug solubility in the digestive environment, chemical stability, lipophilicity, ionizability, and pharmaceutical form.

**Figure 2:**
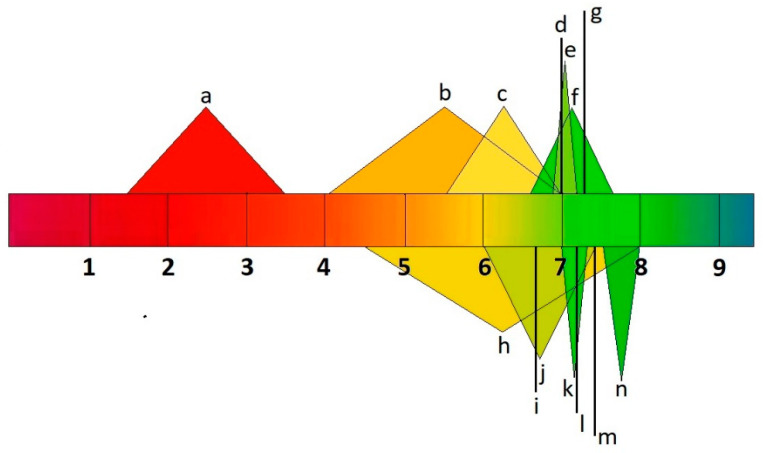
pH values of human body fluids a: stomach; b: small intestine; c: large intestine; d: liver; e: muscle; f: uterus; g: testis; h: bladder; i: lungs; j: saliva; k: kidneys; l: brain, heart, and spleen; m: bone; n: pancreas.

A drug must be soluble in the intestinal environment to be absorbed across biological membranes. Additionally, the ability of a drug to penetrate the membrane can be altered by its ionic form, which depends on the environment’s pH [35]. Drug molecules can ionize over different pH ranges, and the acid–base dissociation constant (pKa) is adopted as an universal measure of ionization. The pH range ϵ (2–12) indicates the point at which 63% of drugs ionize, according to the World Narcotics Index. About 43% and 12% of drugs, respectively, contain a single primary or acid center. The ionization process can significantly impact the properties of drug absorption, distribution, metabolism, excretion, and toxicity in vivo. Changing a drug’s ionic form (Figure 2) can affect its action, absorption, and therapeutic efficacy, depending on the environment’s pH [36,37].

The bioavailability of drugs can be significantly affected by various biological processes occurring in the body, such as gastric acidity, organ blood flow, digestive enzyme activity, intestinal microflora, and biological barriers. Gastric acidity, for example, may influence drug distribution and solubility. Furthermore, the activity of digestive enzymes in the gut can either reformulate the drug or reduce its availability, influencing its effectiveness [38]. Biological barriers, such as the intestinal cell membrane and blood–brain barrier, can limit the penetration of a drug into the bloodstream [39]. Biological barriers protecting the system against pathogens complicate drug delivery and distribution [40]. The adult brain possesses five barrier interfaces that regulate molecular movement into the brain parenchyma. These are the blood–brain barrier (BBB) the blood–cerebrospinal fluid barrier (BCSFB) [41], the blood–arachnoid barrier (BAB) [42], the circumventricular organs (CVOs) [43], and the ependyma [44]. The blood–brain barrier (BBB) is created by a tight structure of endothelial cells (ECs) joined together by protein couplings. These cells line the cerebral microvessels, separating the blood from the brain’s interstitial fluid [45]. The choroid plexus epithelium is between the blood and the ventricular cerebrospinal fluid (CSF) and forms the blood–CSF barrier. The epithelium between the blood and the subarachnoid CSF forms the arachnoid barriers. These three barrier layers limit and regulate molecular exchange at the interface between the blood and neural tissue or its fluid spaces. [46]. The inherent function of biological barriers impedes drug delivery and uptake, preventing effective therapeutic interventions. Biological barriers hinder treatment options and reduce the bioavailability of drugs in areas protected by barriers, which can ultimately lead to increased drug resistance [47].

Pharmaceutical factors encompass the technologies used to create a drug. These include drug formulation, excipients, formulation methods, and drug release techniques [48]. The main objective of any drug delivery system (DDS) is to maintain the desired therapeutic effect by providing and sustaining adequate drug concentration at the target site in the body. This involves enhancing drug efficacy; resolving issues related to solubility, low bioavailability, and poor in-body distribution; and minimizing side effects. Since 2015, significant progress has been made in the research on drug delivery methods and related fields, such as drug science, material science, and biomedical science. This progress has led to the development of superior drug forms [49].

The drug formulation process often involves combining inactive ingredients and additional substances with APIs to produce drug products with specific characteristics. Improving this process to achieve an optimal drug formulation can involve various objectives such as increasing efficacy, extending the duration of therapeutic effects, reducing adverse effects, prolonging the shelf life of active ingredients, and enhancing compatibility with patient intake patterns [1]. APIs can be formulated using different material combinations, including neutral boosters such as polymers, lipids, surfactants, and other active ingredients, depending on the desired delivery method and specific application requirements. Such formulations are made possible by utilizing various types of delivery systems, including different kinds of microparticles (MPs), nanoparticles (NPs), and complex multi-component systems [2,3,4,5]. Typical practices involving these delivery mechanisms are often evolving, resulting in the development of drug products in various forms, such as solids, liquids, or non-oral administration methods [50].

**Figure 3:**
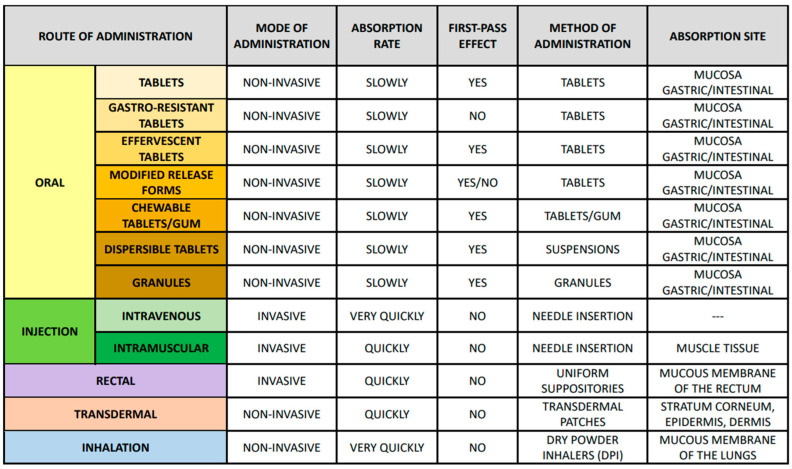
Routes of administration, including their characteristics, location and absorption rate, first pass effect, and method of administration.

Drug forms such as tablets, capsules, granules, powders, suspensions, solutions, emulsions, inserts, ointments, inserts, aerosols, patches, and transdermal systems affect the drug’s dissolution rate and absorption [51]. Excipients, such as binders, solvents, and stabilizers, can affect a drug’s bioavailability through interactions with the drug or changes in its solubility [52]. Drug release techniques can control the active ingredient’s release rate, which affects its availability and action [48]. Acetylsalicylic acid, commonly known as aspirin, is available in various administration forms. It can be taken orally as tablets in enteral, enteric-coated, effervescent, and controlled-release forms. Additionally, there are also chewable versions, in tablet or gum form, and there is also the possibility of preparing it in a suspension or as granules. The drug can also be administered in uniform rectal suppositories, through injection, by absorption via the skin, or as an inhalation. The effectiveness and action of the drug depend on the selected mode of administration and the form of the drug used (Figure 3) [53,54].

Patient factors refer to individual patient characteristics that can affect the bioavailability of a drug [55]. These include age, gender, genotype, health status, and diet. The drug absorption, metabolism, and elimination processes may differ depending on age. Children and the elderly may exhibit differences in metabolic enzymes, renal function, and blood flow, which can affect the bioavailability of drugs. Aminoglycosides are an example of such a drug. Depending on the age of the patients, they are used at different administration intervals: every 8 h in older children, every 12 h in newborns, and every 24 h in premature infants [56].

Gender can significantly affect how drugs are absorbed, metabolized, and eliminated in the body. Hormonal differences and variations in body composition can also affect how drugs are processed in the body. Additionally, genetic variations can impact the activity of enzymes or transporters involved in drug bioavailability and elimination processes. These factors can lead to differences in the way individuals respond to medications. Between 2003 and 2016, it was noted that selective serotonin reuptake inhibitors caused side effects in 68% of women and more frequent severe reactions in 31.6% of men. These side effects were typically dose-dependent and characteristic of the product. Of the 59 frequently reported adverse reactions, 16 were more common in women and 4 in men [57,58].

Various conditions, such as liver, kidney, gastrointestinal, or heart disease, can affect drug metabolism and elimination, having important implications for treating many conditions [55]. Disorders of the function of these organs can affect the bioavailability of drugs, meaning that the amount of API available in the body can be altered [24,59]. This is particularly important in treating HIV infection [60].

The protein known as P-glycoprotein (P-gp) plays a critical role in creating barriers within cells, particularly in the endothelial cells of the blood vessels. Its primary role is to prevent the entry of various substances, including drugs, into neural tissue by removing them from endothelial cells and returning them into the bloodstream. P-gp is a multidrug transporter that can recognize many compounds with different chemical structures and molecular weights (ranging from 330 to 4000 Da) [61]. It can transport hydrophobic and inert substances, as well as cations, but it cannot transport anions. The log *p* value of approximately 2.2 for DTG [62] indicates that it is only partially subject to bioaccumulation due to its moderate hydrophobicity [63]. P-gp is a protein crucial in transporting substances into and out of cells. Dolutegravir (DTG) is a substance for which P-gp is particularly important. Studies have shown that when DTG enters endothelial cells from the blood, it is pumped back into the bloodstream due to P-gp activity. However, disruption of the blood–brain barrier caused by HIV can lead to the dysfunction of P-gp, making it easier for drugs like DTG to penetrate brain tissues. This can result in higher concentrations of DTG in the brain, leading to unwanted side effects such as insomnia and headaches [64]. It is important to note that P-gp is present in tissues with a secretory function, such as the small intestine, liver, and kidney. If there is a pathological dysfunction of the P-gp protein, it can result in increased symptoms of dysfunction in these tissues. Recent studies reveal that P-gp triggers the production of effector T cells after viral infection. Additionally, in cases of bacterial invasion, P-gp plays a protective role against memory T cells [65]. This has significant implications for disease progression in HIV infections.

Specific dietary components, other medications taken, and whether or not the drugs are taken with food, for example, can affect absorption processes. Some drugs may be better absorbed in fats [66]. Patients taking multiple drugs may experience interactions at the metabolic enzyme or transporter level, affecting drug bioavailability and elimination [67].

## 4. Methods for Assessing Drug Bioavailability

Three main categories of methods are used to assess drug bioavailability—in vitro methods, in vivo methods, and new techniques and tools. 

In vitro methods are laboratory-based and involve studying drug bioavailability under controlled conditions outside the living organism. These methods enable researchers to study drug absorption, metabolism, and transport processes and to evaluate the impact of physicochemical factors on drug bioavailability [68]. There are different in vitro methods used for drug testing. One such method is drug solubility testing, in which the solubility of a drug is measured in various environments, including the use of buffer solutions with different pH levels. This test helps determine how well a drug dissolves and is absorbed in the digestive environment [69]. Another method is membrane diffusion testing, in which artificial membranes or tissue fragments are used to study how a drug penetrates a biological barrier and to determine its absorption rate [70]. Studies determining drug permeation across the biological barrier often consider the P-pg protein’s critical influence on drug distribution and elimination from the body. P-gp protein plays a vital role in forming cellular barriers and acts as a membrane protein transporter, actively removing harmful substances from cells. It localizes primarily in organs crucial for the distribution and excretion of drugs from the body, such as the brain, placenta, liver, intestine, and kidneys. The presence of P-gp protein in capillary endothelial cells is significant in the context of barrier formation, i.e., the blood–brain barrier, the blood–nuclear barrier, or the blood–placenta barrier [61]. It forms a trans-membrane unidirectional efflux pump using ATP to actively transport substances out of cells against their concentration gradients. P-gp has also been shown to be strongly involved in multidrug-resistant diseases [71].

Cell cultures are another example in which human or animal cells are used to simulate processes such as drug absorption, metabolism, and transport. These cultures can also be used to examine the effects of digestive enzyme activity or transporters on bioavailability [72]. Narrow liver microsomes containing microsomal enzymes accurately represent the liver’s metabolic activity, with various applications. Studying drug metabolism using hepatic microsomes allows for the estimation of how a drug may be metabolized before it is eliminated from the system [73].

Through in vivo techniques, the bioavailability of a drug in a living system can be investigated. These methods consider the entire bioavailability process, including drug interactions, metabolism, elimination, and patient response. In vivo methods involve administering drugs to patients or animals and then analyzing samples of blood, urine, or other body fluids to determine the concentration of the drug over time [74]. An example of an in vivo method is a pharmacokinetic study involving the determination of the kinetics of a drug in the body, i.e., its absorption, distribution, metabolism, and elimination. The concentration of the drug in various tissues and body fluids is monitored at specific time intervals to understand how the body processes the drug [75]. A bioequivalence study is another example that compares the bioavailability of the original drug with its generic counterpart. This study involves comparing the concentration of the two drugs under examination in the blood or other body fluids. These studies aim to determine whether two drug preparations are pharmaceutically equivalent and exhibit similar therapeutic effects [76]. In vivo procedures include pharmacodynamic studies that evaluate the way a drug impacts the human organism following delivery. These studies are based on the determination of drug concentrations in target tissues, their interaction with the receptors, and the biological effects. They aid in the evaluation of a drug’s therapeutic effect and the way in which it is influenced by bioavailability [77]. An example of such studies is the analysis of drug interactions to determine if one drug affects the absorption, metabolism, and elimination of another drug. This is especially relevant in cases of polytherapy and potential drug interactions [78].

With the advancements in science and technology, new methods and tools for evaluating drug bioavailability are being developed. Medical imaging techniques, such as computed tomography (CT), magnetic resonance imaging (MRI), and positron emission tomography (PET), are some of the techniques that are currently being developed. These techniques enable the monitoring and tracking of drug distribution in the body, allowing for the observation of the drug’s path post-administration and the assessment of its concentration in various tissues and organs [79]. Other advanced techniques include pharmacogenetic studies, which deal with the impact of genetic differences on drug responses. They allow for the determination of the effect of genetic polymorphism in metabolic enzymes and transporters on drug bioavailability in different individuals [80]. Pharmacokinetic modeling, or computer simulation, has become a valuable medical tool that uses mathematical models and algorithms to predict drug performance in living organisms, considering factors like dosage, absorption, transport rates, and enzyme concentrations, thereby optimizing treatment efficacy [81].

## 5. Drugs with Poorly Described Bioavailability

Drugs with poorly described bioavailability are those for which there is limited information regarding the ADME process in the body. There may be various reasons for the lack of detailed data on drug bioavailability [21]. In this context, four distinct groups of drugs can be identified: (1) drugs with complex metabolism and elimination, (2) drugs with limited solubility, (3) drugs with specific absorption, and (4) other cases. Drugs with complex metabolism and elimination undergo intricate chemical metabolism and removal processes, influenced by factors such as interactions with other drugs, differences in gene-type metabolism, diseases, and the overall health of the patient [34].

Warfarin is a drug used for preventing and treating thrombosis. However, its metabolism is quite complex. It is mainly metabolized in the liver by cytochrome P450 enzymes through a series of steps involving hydroxylation, reduction, and conjugation. After metabolism, the drug is eliminated from the body as metabolites through the kidneys. However, the activity of cytochrome P450 enzymes varies widely due to genetic differences and interactions with other drugs and food. As a result, warfarin’s bioavailability and concentration may differ between patients [82,83].

Carbamazepine is a medication used to treat epilepsy, trigeminal neuralgia, and bipolar affective disorder. Certain enzymes, particularly CYP3A4 and CYP2C9 isoenzymes, metabolize the drug in the liver. Its metabolites are excreted in both urine and feces. Carbamazepine’s metabolism and bioavailability can be significantly affected by interactions with other drugs that inhibit or induce the cytochrome enzymes [84,85].

Digoxin is a medication used to treat heart failure and certain cardiac arrhythmias. Although it is primarily metabolized in the liver, it is eliminated from the body mainly through renal excretion. The metabolism of digoxin is complex, with glucuronidation being the primary metabolic pathway. However, the therapeutic window for digoxin is narrow, which means that even small changes in bioavailability and elimination can cause toxicity or a lack of effectiveness [86,87].

Drugs with low water solubility show difficulty dissolving in body fluids, which affects their bioavailability and therapeutic effectiveness due to the hydrophobicity of the drug or the formation of complexes with other substances [88,89]. Analgesics like diclofenac possess limited solubility in water, which adversely affects their absorption from the gastrointestinal tract and therefore, their bioavailability. Various strategies can be employed to increase the solubility and improve the absorption of these drugs. These may include modifying the formulation to obtain a more soluble form or using specific carriers that facilitate drug delivery to the site of action [90]. Certain antifungal medications, including itraconazole, exhibit a restricted capacity to dissolve in water, reducing their absorption in the gastrointestinal tract. To overcome this issue, solubility-enhancing substances are administered, or suitable formulations are developed to enhance the effectiveness and bioavailability of these drugs [91]. Paclitaxel is a common anticancer drug used to treat various malignancies in humans. However, it shows limited solubility in water, which makes it difficult to administer and absorb. To overcome this challenge, unique formulations enhance its solubility and delivery to the site of action [92]. Some antiepileptic drugs, such as phenytoin, are challenging due to their limited solubility in water. Phenytoin is hydrophobic and can be difficult to dissolve in body fluids. To address this issue, a microcapsule system is being developed to improve the solubility and bioavailability of phenytoin. This system will not only increase the solubility of phenytoin, but also enhance its absorption [93]. Another drug with limited solubility in water is furosemide, a loop diuretic used to treat hypertension and edema. Due to its hydrophobic properties, furosemide may possess limited bioavailability, but salts and formulations that increase its dispersibility can improve its solubility and bioavailability [94]. 

Drugs with specific absorption are those whose absorption in the gastrointestinal tract depends on specific mechanisms or conditions. These drugs may be subject to interactions with other substances, pH changes, the presence of transporters, or specific absorption processes. One example of such a drug is levothyroxine, a synthetic hormone used to treat hypothyroidism. Its absorption depends on the presence of iodine in the gut. Iodine ions are essential for forming the active thyroid hormone (thyroxine—T4). Consequently, patients taking levothyroxine must take it on an empty stomach and avoid substances such as calcium, iron, or fiber that may affect the absorption of iodine and the drug itself [95]. Fexofenadine is a type of antihistamine medication used to treat allergic rhinosinusitis. It is a substrate for OATP1A2, which is an organic anion-transporting polypeptide 1A2 transporter found in the epithelium of the jejunum. These transporters are responsible for carrying fexofenadine from the jejunum into the bloodstream. Therefore, any drugs that may affect the activity of these transporters can have an impact on the absorption of fexofenadine [96]. Vardenafil is a medication prescribed to treat erectile dysfunction. The drug’s absorption depends on the presence of the CYP3A4 enzyme in the gut. The absorption and bioavailability of vardenafil can be influenced by interactions with other drugs or substances that either inhibit or induce the activity of the CYP3A4 enzyme [97]. Sulfasalazine is a medication used to treat inflammatory bowel diseases like Crohn’s disease and rheumatoid arthritis. Intestinal bacteria break it down into sulfapyridine and 5-aminosalicylic acid (5-ASA). The drug is not well absorbed by the body, since most of it is absorbed in the large intestine, where intestinal bacteria metabolize it [98].

Apart from to the examples outlined above, there are numerous other instances in which drug bioavailability is poorly defined or understood. Herbal treatments, anticancer medications, and novel medications are some examples of these. The bioavailability of certain plant-based drugs, such as herbal dietary supplements, can be inadequately described due to the complexity of their active ingredients, which can vary in their chemical form [99,100]. Hypericin is a natural compound in the St. John’s wort (Hypericum perforatum) plant. It is used for various health conditions, including treating depression and fighting against different viruses. However, the bioavailability of hypericin is not well understood because it is a complex chemical compound that can undergo several transformations in the body. Different forms of hypericin may include various pharmacokinetic properties, as well as bioavailability [99]. Some anticancer drugs, especially those with more complex actions, may exhibit little described bioavailability [101]. Tyrosine kinase inhibitors are an example of drugs that act on different signaling pathways within cancer cells. However, the metabolism and elimination of these drugs can be complex, making it challenging to determine their bioavailability [102]. Limited information on bioavailability may be available when new drugs are marketed. Therefore, pharmacokinetic studies, which measure bioavailability, are required before a drug can be marketed [20]. 

Data on bioavailability may be limited for new drugs that have not undergone comprehensive clinical trials. The absorption, distribution, metabolism, and elimination of a drug are all evaluated in basic pharmacokinetic studies. Nevertheless, data regarding bioavailability are scarce, particularly in large-scale clinical trials with a diverse patient group [21]. An example of a new drug with limited information regarding its bioavailability is tecovirimat (Tpoxx), an antiviral drug that has demonstrated efficacy in animal studies and has been approved by the Food and Drug Administration for the treatment of smallpox, a severe and life-threatening infection caused by the Variola virus of the Orthopoxvirus genus. It belongs to a group of drugs known as orthopoxvirus-specific antivirals. Tecovirimat is an investigational drug and is not currently approved for routine use. It is used in emergencies as part of preparatory measures against smallpox outbreaks [103]. For drugs with little described bioavailability, it is essential to conduct pharmacokinetic and pharmacodynamic studies to better understand how the drug works in the body. These studies can include assessments of absorption, metabolism, elimination, and interactions with other substances. Drug bioavailability factors can be identified through more detailed studies, and strategies can be developed to improve their effectiveness and safety in treating patients [20].

## 6. Challenges and Prospects in Drug Bioavailability Research

The variety of factors affecting bioavailability is large. Interactions with other drugs, gastric pH levels, intestinal flora, and individual genetic differences in patients can alter drug bioavailability. Assessing these factors and their impact on bioavailability is a challenge for researchers. It is necessary to consider and understand these factors when evaluating bioavailability [104].

The testing techniques for assessing bioavailability include in vitro methods, in vivo methods, pharmacokinetic studies, and medical imaging. Each of these techniques exhibits limitations and varying reliability, making it challenging to assess bioavailability accurately. Choosing the proper technique to provide reliable and representative results is essential but can be difficult due to the complexity of bioavailability [105]. 

Researching drug bioavailability involves human clinical trials, which pose a significant challenge in ensuring the safety of study participants and in adhering to ethical standards. It is essential to conduct these trials while following ethical guidelines and protecting patients’ rights, simultaneously striving to obtain the necessary information regarding bioavailability [106]. 

Drug bioavailability research is often expensive and resource-consuming, demanding adequate financial resources and infrastructure [107]. Developing simplified methods to assess bioavailability can alleviate these challenges [108]. 

The bioavailability of drugs can vary depending on different factors, such as the timing of intake, food consumption, and the duration of drug use. Keeping these factors under control can be difficult, as they can impact the results of bioavailability studies and their interpretation [96].

Advancements in bioavailability research have led to the development of new trends and technologies that seek to enhance the effectiveness and safety of drug therapy. These innovations aim to better understand bioavailability and improve its control. Thanks to modern technologies, it is possible to move toward personalized therapy, which targets specific patients and considers individual factors affecting drug bioavailability [91].

**Figure 4:**
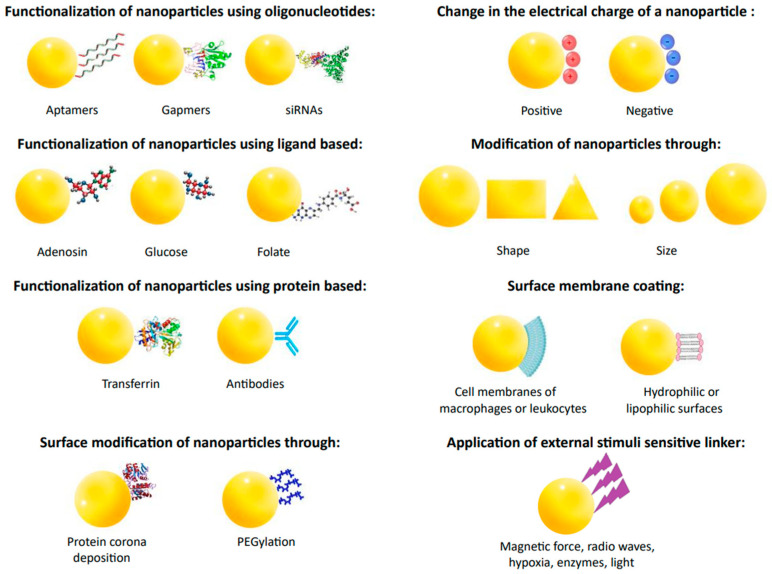
Modification of structural changes in nanoparticles to improve their pharmacokinetics and pharmacodynamics.

Recent advances in nanotechnology and drug delivery technologies are revolutionizing the field of medicine by improving the bioavailability of drugs. By delivering drugs directly to the targeted tissues, drug nanoparticles (shown in Figure 4) can easily cross biological barriers and reach the appropriate site in the body. This approach enables the drugs to act more effectively and efficiently, thus providing better patient results [55,93,109].

Various ligands, antibodies, aptamers, peptides, or proteins can be used to achieve targeted actions to alter the surface of nanoparticles. Examples of ligands include adenosine, folic acid, and glucose. Adenosine targets nanoparticles to tumor co-cells via the A1 receptor. Folate receptors, overexpressed in some cancers, allow for the selective uptake of nanoparticles. Glucose is used due to the increased demand of cancer cells for this sugar. Ligands exhibit advantages over antibodies, i.e., they are cheaper, simpler to combine, and safer. Ligands can be targeted to specific cell sites, making them more effective. Proteins like transferrin activate cellular receptors. Transferrin possesses more receptors in cancer cells, so it is a suitable ligand for targeted treatment. Nanoparticles with transferrin, such as doxorubicin, show anti-tumor effects and reduce side effects. Nanoparticles of human albumin coated with an antibody against transferrin receptors can carry loperamide across the blood–brain barrier. Aptamers are short RNA or DNA oligonucleotides that bind specifically to proteins or biological targets. They are synthesized by an in vitro process called SELEX. Aptamers are less immunogenic and more accessible to produce than antibodies. Gapmers are short antisense oligonucleotides capable of silencing specific RNA. SiRNA is used for gene silencing, which may have applications in anticancer and antiviral therapy. It is exemplified by liposomes delivering siRNAs that block replication of the SARS-CoV-2 virus. Biofilms and mucus layers can affect the degradation of nanoparticles by trapping them in different pore sizes or through non-specific interactions, which can lead to their removal from the epithelial surface. For this reason, nanoparticles are formed in different sizes and shapes. Given the vital role of the microenvironment for nanoparticles, there is a need to design new types of nanoparticles or to modify them to take advantage of this variability. Exogenous triggers, such as near-infrared light, radio waves, or magnetic fields, allow for the controlled delivery of nanoparticles from the outside. Even surrounding nanoparticles with the membranes of immune cells, such as macrophages or leukocytes, improves their effectiveness in targeted cancer cells. Nanoparticles surrounded by cell membranes show significantly enhanced drug activity compared to free drugs [55,110].

Using computer modeling and in silico simulation allows for the prediction of a drug’s bioavailability based on its chemical structure. By analyzing the molecule’s structure, physical properties, and interactions with the body, it is possible to estimate how drugs will behave in the body, including how they are absorbed, broken down, and distributed. This process significantly speeds up the bioavailability assessment and reduces costs associated with in vitro and in vivo studies [111].

The co-crystallization of APIs represents an innovative methodology for modifying the properties of drugs. It can affect the bioavailability of medicines through changes in solubility, controlling the rate and degree of drug absorption and diffusion behavior. The structure of the resulting co-crystals may alter the solution stability of the APIs, thereby affecting their durability [112]. In comparison to the parent pharmaceuticals, the co-crystals exhibit different physicochemical properties, crystal grid energies, and thermodynamic stability due to their distinct crystalline structure. This novel methodology possesses the capacity to enhance the advancement of effective drugs, particularly in cases when medicinal compounds encounter challenges in achieving suitable physicochemical characteristics [113].

In the area of drug bioavailability, the concept of controlled drug release is of major importance in the development of new pharmacological formulations. Controlled drug release systems utilize sophisticated mechanisms to distribute active ingredients in the neighborhood of the target site, hence enabling the control of the concentration of the released compounds over a longer span of time. This particular method has the potential to shape the future of personal therapy. The ability to regulate the rate of drug release from a carrier, such as nanomagnetic carriers, enables the precise administration of drugs tailored to the therapeutic requirements of the body. One method that has been extensively investigated is the utilization of nanomagnetic carriers, which can be directed towards a targeted region, such as tumor cells, in the context of breast cancer treatment. The utilization of a magnetic field enables the control of drug release, thus facilitating targeted drug delivery to an area of interest and limiting potential adverse effects [114]. Drug release control systems are designed to optimize the way in which the body absorbs and uses the API, which can lead to improved bioavailability and therapeutic effectiveness [115].

Conformation composition management refers to the systematic approach employed to govern and oversee the relative proportions of various conformers inside a specific chemical compound. Recent conformation composition management methods include hypercritical fluid technologies, such as the use of supercritical carbon dioxide (scCO_2_). The utilization of supercritical conditions enables the regulation of the relative distribution of various spatial configurations of the pharmaceutical substance. This methodology necessitates the utilization of precise and discerning analytical techniques that enable sufficient regulation and anticipation of the eventual outcome. Notable examples include high-pressure nuclear magnetic resonance (NMR) spectroscopy and techniques such as nuclear Overhauser effect spectroscopy (NOESY). The increased knowledge of molecular structure is made possible by conformation management, and this knowledge can influence various aspects of pharmaceuticals, including their bioavailability [116,117]. 

Serum albumin binding studies are critical for determining the bioavailability and transport of pharmaceuticals in the body. Serum albumins, a type of carrier protein, are critical in the transport of different chemicals, including medicines, through the circulatory system. Drugs that bind to serum albumin are frequently regarded as potential (pro)medicines. The mechanism of drug binding to albumins influences how APIs are carried and distributed to different tissues. This effect is important for drugs that can form complexes with albumins, which can alter their stability and transport in the bloodstream [118].

Research on genetic factors that affect drug bioavailability opens up new possibilities for personalized therapy. Genetic differences in drug metabolism can affect the bioavailability and efficacy of therapy. Therefore, studying genetic polymorphisms related to metabolic enzyme systems, drug transport, or receptors may allow for a better understanding of how the body absorbs and utilizes drugs. This may help optimize drug doses and tailor therapy to patients’ individual needs [119].

Bioavailability research is an essential aspect of medicine and pharmacology. It allows for a better understanding of drugs’ absorption, distribution, and metabolism in the body. This understanding helps to optimize drug doses and administration frequency, resulting in more effective therapy [120]. Improved bioavailability can lead to higher drug concentrations at the target site and a longer duration of action, which contributes to more effective treatment of diseases. Therefore, bioavailability research is multifaceted and crucial to the development of medicine and pharmacology [13].

Studying drug bioavailability enables the personalization of therapy, considering individual factors affecting bioavailability. Drug doses and dosing regimens can be tailored to individual patients by identifying genetic variants, drug interactions, and other factors. This leads to better therapeutic results and minimizes the risk of side effects [24].

Bioavailability is essential in the development of new drugs and formulations. More effective and efficient active ingredients can be designed by understanding the factors that affect bioavailability, such as solubility, chemical stability, and pharmaceutical forms. This leads to innovative drugs with higher bioavailability and a better therapeutic profile [91]. 

For rare and hard-to-treat conditions, the availability of effective drugs may be limited. Consequently, bioavailability research allows for the identification of new drug delivery approaches, such as nanotechnology or genetically targeted therapies. This opens up new therapeutic perspectives for patients with rare diseases [13].

By fine-tuning doses and the frequency of administration, over-medication or toxic effects can be avoided, thereby minimizing the risk of adverse effects. Bioavailability studies can also help identify drugs with better safety profiles and higher bioavailability, while minimizing the risk of side effects [12].

## 7. Conclusions

Bioavailability data for many active compounds is sparse, despite substantial pharmacological study. These extensive pharmacokinetic studies are required for a broad list of drugs [119]. However, such studies are expensive and complicated; thus, few are performed, and therefore, few can be added to the bioavailability dataset [106]. Moreover, the pharmacokinetic characteristics of individuals vary greatly. Age, gender, genetics, health, and other parameters affect drug absorption and transport. However, a number of results obtained from such studies can be utilized to build generalized models showing the action of APIs in the human body [24].

It was established that drug bioavailability depends on administration [121]. Intravenous medications enter the bloodstream directly, while oral pharmaceuticals must pass through the digestive system and may be destroyed or absorbed incorrectly. Many bioavailability details remain undiscovered, despite broad studies for varied dosing techniques [122]. Since not all drug-food interactions are known, the need remains for further thorough studies of this aspect [123].

In regards to drug therapy safety, bioavailability studies determine doses to reduce dangerous blood active component concentrations, identify medication interaction risk factors, and improve safety [124]. Individualized treatment based on genetics, health, age, and drug interactions is possible because these studies disclose internal factors impacting drug absorption, distribution, and metabolism [125].

Medication bioavailability research is essential for treating rare and complex diseases. Understanding the active ingredient absorption derived from tablets, capsules, injections, and patches improves drug development. Understanding bioavailability improves drug use and health care by optimizing prescription design, treating rare disorders, and discovering new formulations [126].

## Data Availability

Not applicable.
